# Risk Factors for Positive Deep Pelvic Nodal Involvement in Patients with Palpable Groin Melanoma Metastases: Can the Extent of Surgery be Safely Minimized? A Retrospective, Multicenter Cohort Study

**DOI:** 10.1245/s10434-015-4602-4

**Published:** 2015-05-27

**Authors:** C. M. C. Oude Ophuis, A. C. J. van Akkooi, H. J. Hoekstra, J. J. Bonenkamp, J. van Wissen, M. G. Niebling, J. H. W. de Wilt, B. van der Hiel, B. van de Wiel, S. Koljenović, D. J. Grünhagen, C. Verhoef

**Affiliations:** Department of Surgical Oncology, Erasmus MC Cancer Institute, University Medical Center Rotterdam, Rotterdam, The Netherlands; Department of Surgical Oncology, Netherlands Cancer Institute – Antoni van Leeuwenhoek, Amsterdam, The Netherlands; Department of Surgical Oncology, Groningen University Medical Center, Groningen, The Netherlands; Department of Surgical Oncology, Radboud University Medical Center, Nijmegen, The Netherlands; Department of Nuclear Medicine, Netherlands Cancer Institute – Antoni van Leeuwenhoek, Amsterdam, The Netherlands; Department of Pathology, Netherlands Cancer Institute – Antoni van Leeuwenhoek, Amsterdam, The Netherlands; Department of Pathology, Erasmus MC, University Medical Center Rotterdam, Rotterdam, The Netherlands

## Abstract

**Background:**

Patients with palpable melanoma groin metastases have a poor prognosis. There is debate whether a combined superficial and deep groin dissection (CGD) is necessary or if superficial groin dissection (SGD) alone is sufficient.

**Aim:**

The aim of this study was to analyze risk factors for deep pelvic nodal involvement in a retrospective, multicenter cohort of palpable groin melanoma metastases. This could aid in the development of an algorithm for selective surgery in the future.

**Methods:**

This study related to 209 therapeutic CGDs from four tertiary centers in The Netherlands (1992–2013), selected based on complete preoperative imaging and pathology reports. Analyzed risk factors included baseline and primary tumor characteristics, total and positive number of inguinal nodes, inguinal lymph node ratio (LNR) and positive deep pelvic nodes on imaging (computed tomography [CT] ± positron emission tomography [PET], or PET − low-dose CT).

**Results:**

Median age was 57 years, 54 % of patients were female, and median follow-up was 21 months (interquartile range [IQR] 11–46 months). Median Breslow thickness was 2.10 mm (IQR 1.40–3.40 mm), and 26 % of all primary melanomas were ulcerated. Positive deep pelvic nodes occurred in 35 % of CGDs. Significantly fewer inguinal nodes were positive in case of negative deep pelvic nodes (median 1 [IQR 1–2] vs. 3 [IQR 1–4] for positive deep pelvic nodes; *p* < 0.001), and LNR was significantly lower for negative versus positive deep pelvic nodes [median 0.15 (IQR 0.10–0.25) vs. 0.33 (IQR 0.14–0.54); *p* < 0.001]. A combination of negative imaging, low LNR, low number of positive inguinal nodes, and no extracapsular extension (ECE) could accurately predict the absence of pelvic nodal involvement in 84 % of patients.

**Conclusions:**

Patients with negative imaging, few positive inguinal nodes, no ECE, and low LNR have a low risk of positive deep pelvic nodes and may safely undergo SGD alone.

Patients with clinically palpable nodal metastases of cutaneous melanoma in the groin have a poor prognosis. Balch et al. reported a 5 year overall survival (OS) rate of 59 % for stage IIIB melanoma in the 2009 American Joint Committee on Cancer (AJCC) melanoma staging system analysis.^1^ Reported 5 year OS rates for the subgroup of patients with palpable groin metastases ranged from 52 % for superficial involvement to 12 % for deep involvement.^2^–^7^

Standard of care for these patients consists of therapeutic lymph node dissection (TLND),^2^^,^^8^–^10^ and there is ongoing debate as to whether this should consist of either a combined superficial and deep groin dissection (CGD) or whether a superficial groin dissection (SGD) would suffice.

Several cohort studies indicate no difference in survival between these two procedures, and patients may benefit from SGD alone if no positive deep pelvic nodes are present on preoperative imaging.^2^^,^^8^^,^^10^^–^12

Since the estimated prevalence of positive deep pelvic nodes in patients with palpable inguinal lymph nodes is 30 %, the majority of patients undergoing CGD may not benefit from deep groin dissection (DGD).^6^^,^^12^ As CGD is a more extensive procedure than SGD, the risk of morbidity is potentially higher.^6^ A clear need exists to select those patients who can be safely spared a DGD in the absence of deep pelvic nodal involvement.^10^^,^^11^^,^^13^–^15^

Preoperative imaging techniques such as computed tomography (CT) and positron emission tomography (PET) form a valuable adjunct to staging. Up to 27 % of patients presenting with palpable lymph node metastases have synchronous distant metastases at preoperative PET/CT, which changes the indication for surgery into palliative resection and/or systemic therapy.^16^ Additionally, imaging provides assessment of suspicious deep pelvic nodes prior to surgery. High positive (PPV) and negative predictive value (NPV) have been achieved by Allan et al. (100 and 86 %, respectively).^3^ Other series reported PPVs and NPVs of 40–60 %, which is too low to confirm or reject the presence of positive deep pelvic nodes based on preoperative imaging alone.^2^^,^^17^^,^^18^ Once suspicious deep pelvic nodes are detected on preoperative imaging, one cannot ignore their presence and CGD is highly recommended. The absence of suspicious deep pelvic nodes on imaging does not rule out deep pelvic nodal involvement. Once imaging has been performed, the focus should be on identification of further risk factors for positive deep pelvic nodes.^2^^,^^7^^,^^11^^,^^15^^,^^17^–^21^

## Patients and Methods

### Patients

This retrospective, multicenter cohort study described 209 therapeutic CGDs performed at four tertiary melanoma centers in The Netherlands between 1992 and 2013. Patient selection was based on the presence of a palpable nodal metastasis to the groin, complete pathology reports of the performed CGD (i.e. clearly describing the dissected lymph nodes as inguinal or iliac, including obturator area), and preoperative imaging (CT, PET, or PET/CT). Patients without imaging, with prior lymph node dissections in the groin area, or with isolated limb perfusion or positive sentinel node(s) as an indication for CGD were excluded. Analyzed preoperative imaging modalities were CT scan, PET, and combined PET with low-dose CT (PET/CT).

All patient characteristics were obtained from medical records and collected in a database for the current study, according to local Institutional Review Committee guidelines and national legislation.

### Surgical Procedure

CGD was performed either via two separate transverse incisions or via an inguinal ellipse-shaped incision extending cranially according to local preferences per center, as described in detail elsewhere.^6^^,^^22^

### Pathology

CGD pathology reports were considered adequate when a clear description was given of the total number of inguinal nodes as well as the number of tumor-positive inguinal nodes, and a similar description was given of the number of dissected deep pelvic nodes (iliac nodes and obturator nodes) and the number of tumor-positive deep pelvic nodes.

### Statistics/Data Analysis

Patients were divided into two categories based on deep pelvic nodal status—positive or negative. Univariable *χ*^2^ tests were performed to test for significant differences in prevalence of sex, primary tumor located on the trunk, primary tumor stage (T1–T4), ulceration, and inguinal extracapsular extension (ECE). Nonparametric tests were performed to test for differences in age, median Breslow thickness, total number of inguinal nodes and number of positive inguinal nodes, total number of excised nodes and number of positive nodes, total number of deep pelvic nodes, number of positive deep pelvic nodes, and LNR. Sensitivity, specificity, PPV, NPV, and accuracy were calculated for all imaging modalities using the number of true positives (TP), false positives (FP), true negatives (TN), and false negatives (FN).

Differences in baseline characteristics were tested using univariable logistic regression analysis, multivariable models were calculated using variables significant at univariable analysis, and binary logistic multivariable regression analyses were performed to test for independent predictors of deep pelvic nodal involvement.

Ridge regression analysis was performed to exclude the influence of multicollinearity in a prediction model based on independent predictive variables. An area under the receiver operating characteristic curve (AUC) was calculated for the model. The AUC indicates the probability that patients with observed positive deep pelvic nodes had a higher predicted probability than patients with observed negative deep pelvic nodes, providing information about the predictive value of the model.

All statistical analyses, with the exception of Ridge regression, were performed using SPSS version 21.0 (released 2012; IBM Corporation, Armonk, NY, USA). Ridge regression was performed using RStudio (RStudio Inc., Boston, MA, USA). An * α* < 0.05 was considered significant.

## Results

### Patients

Table 1 provides an overview of baseline characteristics. The majority of patients (*n* = 201, 96 %) had palpable stage IIIB disease, and eight patients (4 %) had stage IV disease. Median Breslow thickness was 2.10 mm (interquartile range [IQR] 1.4–3.4 mm), 12 patients had a history of negative sentinel node, and median follow-up was 21 months (IQR 11–46 months).

**Table 1:** Baseline characteristics

Characteristic	Total [*n* = 209 (100 %)]	Negative pelvic nodes [*n* = 135 (65 %)]	Positive pelvic nodes [*n* = 74 (35 %)]	*p* value
Sex				
Female	114 (54)	76 (56)	38 (51)	0.49
Male	95 (46)	59 (44)	36 (49)	
Age [years; median (IQR)]	57 (45–65)	55 (46–65)	59 (44–65)	0.63
Center				
1	60 (29)	42 (31)	18 (24)	0.21
2	57 (27)	38 (28)	19 (26)	
3	24 (12)	11 (8)	13 (18)	
4	68 (32)	44 (33)	24 (32)	
Tumor stage				
T1	22 (11)	9 (7)	13 (18)	0.16
T2	57 (27)	36 (27)	21 (28)	
T3	60 (29)	39 (29)	21 (28)	
T4	30 (14)	22 (16)	8 (11)	
Unknown primary	10 (5)	8 (6)	2 (3)	
Missing	30 (14)	21 (15)	9 (12)	
Ulceration				
Absent	125 (60)	74 (55)	51 (69)	0.16
Present	54 (26)	38 (28)	16 (22)	
Missing	30 (14)	23 (17)	7 (9)	
Clark level^a^				
II	1 (0.5)	0 (–)	1 (1)	0.29
III	35 (17)	22 (16)	13 (17)	
IV	84 (40)	54 (40)	30 (41)	
V	13 (6)	11 (8)	2 (3)	
Missing	76 (36.5)	48 (36)	28 (38)	
Location				
Leg	166 (80)	106 (79)	60 (81)	0.62
Trunk	28 (13)	17 (13)	11 (15)	
Unknown primary	10 (5)	8 (6)	2 (3)	
Missing	5 (2)	4 (3)	1 (1)	
Histology				
SSM	67 (32)	44 (32)	23 (31)	0.24
NM	37 (18)	28 (21)	9 (12)	
Other	15 (7)	9 (7)	6 (8)	
Unknown primary	10 (5)	8 (6)	2 (3)	
Missing	80 (38)	46 (34)	34 (46)	
No. of nodes [median (IQR)]				
Inguinal	10 (7–13)	10 (7–13)	9 (7–12)	0.54
Deep	6 (4–10)	6 (4–9)	8 (5–11)	0.039^b^
Total	17 (13–22)	17 (13–21)	17 (14–22)	0.39
No. of positive nodes [median (IQR)]				
Inguinal	2 (1–3)	1 (1–2)	3 (1–4)	<0.001^b^
Deep	0 (0–1)	0 (0)	2 (1–3)	<0.001^b^
Total	2 (1–4)	1 (1–2)	5 (3–7)	<0.001^b^
LNR [median (IQR)]	0.20 (0.11–0.33)	0.15 (0.10–0.25)	0.33 (0.14–0.54)	<0.001^b^
Inguinal ECE				
No	134 (64)	94 (70)	40 (54)	0.025^b^
Yes	75 (36)	41 (30)	34 (46)	

Data are expressed as *n* (%) unless otherwise specified

*IQR* interquartile range, *T1* Breslow < 1.00 mm, *T2* Breslow 1.01–2.00 mm, *T3* Breslow 2.01–4.00 mm, *T4* Breslow > 4.00 mm, *LNR* inguinal lymph node ratio, *ECE* extracapsular extension

^a^Clark levels II and III were combined for the *χ* ^2^ test

^b^Significant, *p* < 0.05, calculated using *χ* ^2^ and non-parametric tests

### Imaging and Pathology

Four patients underwent both CT and PET/CT; they were scored as PET/CT since the additional information obtained from PET/CT was used for the final determination of clinical node status. Predictive accuracy per imaging modality is shown in Table 2. The different imaging modalities were used equally between the two groups (i.e. positive or negative deep pelvic nodes).

**Table 2:** Identification of positive deep pelvic lymph nodes using preoperative imaging techniques (*n* = 209)

	CT (%) [*n* = 67]	CT and/or PET (%) [*n* = 57]^a^	PET/CT (%) [*n* = 85]^b^
Sensitivity	57	36	61
Specificity	93	94	83
PPV	80	73	68
NPV	83	70	79
Accuracy	82	70	75

*CT* computed tomography, *PET* position emission tomography, *PET/CT* combined PET and low-dose CT, *PPV* positive predictive value, *NPV* negative predictive value

^a^Thirteen patients underwent PET alone

^b^Four patients also underwent separate CT

### Logistic Regression Analysis

Variables significantly different on univariable analysis (Table 1) were included in multivariable binary logistic regression analyses. LNR and number of positive inguinal lymph nodes were assessed in separate models due to evident multicollinearity. The remaining significant independent predictors were suspicious deep pelvic nodes on imaging (odds ratio [OR] 9.64, 95 % CI 4.35–21.3, *p* < 0.001), increasing LNR (OR 34.2, 95 % CI 5.47–214, *p* < 0.001), the presence of ECE (OR 2.13, 95 % CI 1.01–4.48, *p* = 0.046) and, in a separate multivariable model without LNR, increasing number of positive inguinal lymph nodes (OR 1.27, 95 % CI 1.06–1.53, *p* = 0.010).

### Subgroup Analysis Negative Imaging

Suspicious deep pelvic nodes on imaging were highly predictive for positive deep pelvic nodes. A subgroup of 155 patients without suspicious deep pelvic nodes on imaging was selected for further analysis of additional risk factors for positive deep pelvic nodes. Thirty-five of these patients (23 %) had positive deep pelvic nodes at histopathological examination with hematoxylin and eosin (H&E) staining, i.e. imaging was FN. Univariable analysis results are displayed in Table 3. Multivariable analysis was performed, including all significant variables assumed to be predictive for deep pelvic nodal status: number of positive inguinal nodes, LNR, and ECE status. Evident multicollinearity was observed.

**Table 3:** Baseline characteristics for patients with negative preoperative imaging

Characteristic	Total (*n* = 155)	Pelvic nodes− (*n* = 120)	Pelvic nodes+ (*n* = 35)	*p* value
Sex				
Female	83 (54)	67 (56)	16 (46)	
Male	72 (46)	53 (44)	19 (54)	0.29
Age [years; median (IQR)]	56 (45–64)	55 (46–65)	57 (44–64)	0.99
Center				
1	44 (28)	38 (32)	6 (17)	
2	48 (31)	35 (29)	13 (37)	
3	18 (12)	10 (8)	8 (23)	
4	45 (29)	37 (31)	8 (23)	0.17
Breslow [median (IQR)]	2.10 (1.40–3.25)	2.20 (1.45–3.55)	1.90 (1.15–2.80)	0.11
Tumor stage				
T1	14 (9)	8 (7)	6 (17)	
T2	45 (29)	33 (28)	12 (34)	
T3	44 (28)	37 (31)	7 (20)	
T4	23 (15)	19 (16)	4 (11)	
Unknown primary	9 (6)	7 (6)	2 (6)	
Missing	20 (13)	16 (13)	4 (11)	0.38
Ulceration				
Absent	90 (58)	67 (56)	23 (66)	
Present	44 (28)	36 (31)	8 (23)	
Missing	21 (14)	17 (13)	4 (11)	0.34
Clark level^a^				
II	1 (0.6)	0 (–)	1 (3)	
III	25 (16)	20 (17)	5 (14)	
IV	65 (42)	49 (41)	16 (46)	
V	11 (7)	11 (9)	0 (–)	
Missing	53 (34)	40 (33)	13 (37)	0.070
Location				
Leg	118 (76)	93 (78)	25 (71)	
Trunk	23 (15)	16 (13)	7 (20)	
Unknown primary	9 (6)	7 (6)	2 (6)	
Missing	5 (3)	4 (3)	1 (3)	0.81
Histology				
SSM	52 (34)	40 (33)	5 (14)	
NM	31 (20)	26 (22)	12 (34)	
Other	10 (6)	8 (7)	2 (6)	
Unknown primary	9 (6)	7 (6)	2 (6)	
Missing	53 (34)	39 (32)	14 (40)	0.86
No. of nodes [median (IQR)]				
Total	17 (13–21)	17 (13–21)	17 (14–22)	0.42
Inguinal	10 (8–12)	10 (8–13)	9 (8–12)	0.69
Deep	6 (4–9)	6 (4–9)	7 (4–11)	0.15
No. of positive nodes [median (IQR)]				
Total	2 (1–4)	1 (1–2)	5 (3–6)	<0.001^b^
Inguinal	1 (1–3)	1 (1–2)	3 (1–4)	<0.001^b^
Deep	0 (0)	0 (0)	2 (1–2)	<0.001^b^
LNR [median (IQR)]	0.17 (0.11–0.31)	0.21 (0.10–0.25)	0.33 (0.13–0.50)	0.001^b^
ECE inguinal				
No	96 (62)	79 (66)	17 (49)	
Yes	59 (38)	41 (34)	18 (51)	0.075

Data are expressed as *n* (%) unless otherwise specified

*IQR* interquartile range, *T1* Breslow < 1.00 mm, *T2* Breslow 1.01–2.00 mm, *T3* Breslow 2.01–4.00 mm, *T4* Breslow > 4.00 mm, *LNR* inguinal lymph node ratio, *ECE* extracapsular extension

^a^For the *χ* ^2^ test, Clark II and III were combined

^b^Significant (*p* < 0.05)

**Fig. 1:**
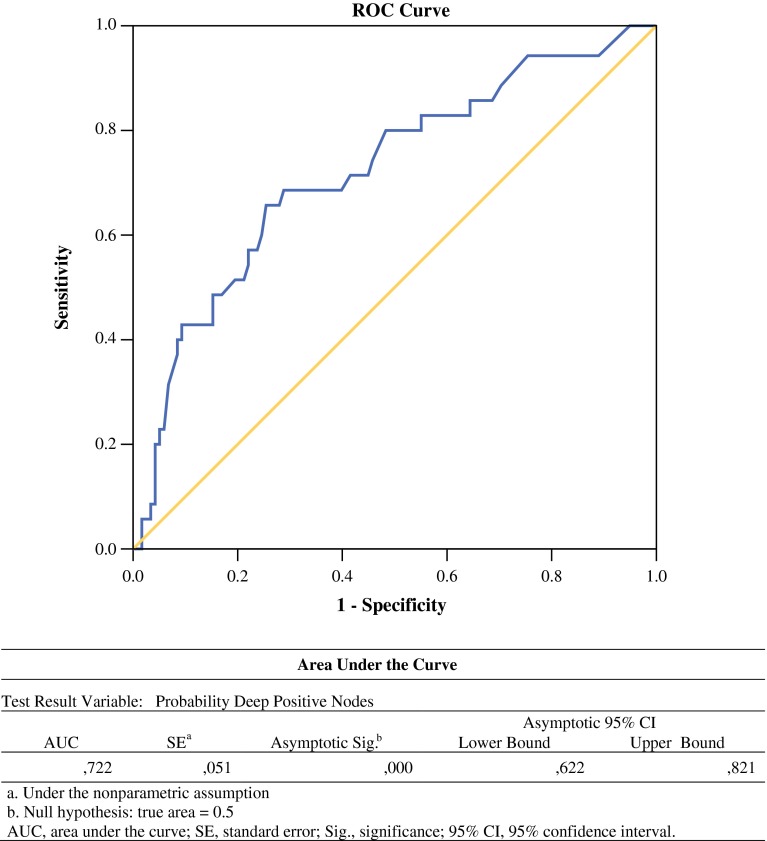
ROC curve for prediction model probability positive deep nodes. *ROC* receiver operator characteristics

To overcome this problem, a predictive Ridge logistic regression analysis was performed. Only LNR remained as a significant independent predictor for positive deep pelvic nodes (*p* = 0.014). The number of positive inguinal lymph nodes and ECE were chosen to remain in the model as contributing covariates as these were thought to be of substantial additional clinical relevance. A receiver operating characteristic (ROC) curve of the predicted probabilities for positive deep pelvic nodes was created, displaying a fair AUC of 0.72 (AUC values ranged between 0 and 1, where high scores are indicative of high accuracy) (Fig. 1). The optimum cut-off value for the predicted probability of the model (i.e. the probability at which the model outcome correctly identifies an observed positive patient as positive) was chosen based on high specificity in order to minimize FN outcomes. Corresponding probability cut-off value and sensitivity were deduced from the ROC curve. For a specificity of 90 %, the cut-off value for a positive test outcome was a probability for positive deep pelvic nodes of 32 % or more. Sensitivity was 43 %, PPV 50 %, NPV 84 %, and overall accuracy of this model was 77 %.

## Discussion

In this CGD cohort, 35 % of all patients had deep pelvic nodal involvement, which is in line with the literature.^10^^,^^11^^,^^13^^–^15 This study analyses risk factors to identify deep pelvic nodal involvement, with imaging being a strong predictor. Our prediction model might lower the rate of CGD without positive pelvic nodes, and minimizes the number of FN outcomes after imaging.

### Imaging

The imaging modalities used in this study are fair in correctly predicting positive deep pelvic nodes; however, a considerable number of patients have FP imaging (20–32 %), and we can only speculate on the possible causes of FP imaging. This might be partially explained by a small group of patients undergoing diagnostic excision biopsy of the palpable lymph node prior to imaging, which might cause lymph node enlargement in the pelvic area. Another cause may be the inevitable interobserver variability in radiology. Improvement of imaging techniques over time may have altered the number of FP lymph nodes detected during the present study period.

NPVs of the preoperative imaging techniques performed in the current study range between 70 and 83 %, leaving a substantial proportion of 23 % (17–30 %) of patients to be falsely diagnosed with negative deep pelvic nodes. Several studies have reported on the NPV of CT, and although high NPVs have been described by Allan et al. and Van der Ploeg et al. overall reported values ranged considerably.^2^^,^^3^^,^^6^^,^^17^^,^^18^ Ongoing development of the newest imaging techniques, such as the use of a melanoma-specific PET tracer ([18F]ICF01006), may enhance the accuracy of imaging and subsequently decrease the FN rate.^23^

### Predictive Factors

Predictive factors for deep pelvic nodal involvement found in the current study are inguinal nodal status as defined by the number of positive inguinal nodes and LNR, inguinal ECE, and suspicious deep pelvic nodes on preoperative imaging, which is concordant with the literature.^2^^,^^7^^,^^11^^,^^15^^,^^17^–^21^ These risk factors may be applied to select patients for SGD, in addition to imaging without suspicious deep pelvic nodes. A hypothetical two-stage approach would be when preoperative imaging is negative, patients first solely undergo an SGD. The pathology results can then be used to determine the risk of occult positive deep pelvic nodes, and a decision can be made on whether to perform an additional DGD or not. The fact that patients must undergo two separate operations is a drawback, but this way a DGD can be spared in 126 of all patients (60 %).

### Patient Selection

Standard CGD for palpable stage III melanoma shows that 135 of 209 deep pelvic groin dissections (65 %) have been performed in the absence of pelvic nodal metastases.

Use of preoperative imaging alone for selection between CGD and SGD would reduce the number of CGDs from 209 to 54. The remaining 155 patients would undergo SGD alone. Thirty-five of these 155 patients undergoing SGD alone were FN (FN rate 23 %) and would possibly be undertreated (i.e. undergoing no DGD).

Better patient selection is necessary in the negative imaging group as a potential decrease in the number of FNs will make patient selection safer. This formed the rationale for the prediction model, which is based on 153 patients (155—2 patients with missing data) with negative imaging. Using this model, 124 of 153 patients would undergo SGD alone, and FN rates would be reduced to 20 of 124 patients (FN rate 16 %).

Concluding, this model forms an adjunct to the use of preoperative imaging as a selection tool for SGD or CGD, both drastically minimizing the number of patients without affected pelvic nodes undergoing a DGD, and controlling the number of patients with affected pelvic nodes potentially being undertreated by not undergoing a DGD.

The 16 % FN rate of this model is still considerable. Although surgery forms the cornerstone of melanoma treatment, one may question the role of DGD in the current era of upcoming effective systemic treatments. On one hand, the majority of patients undergoing standard CGD for palpable groin metastases have negative deep pelvic nodes, while on the other hand there is evidence to assume that positive deep pelvic nodes may merely be a biomarker for stage IV disease as survival rates depend on deep pelvic nodal status rather than extent of surgery.^6^^,^^8^^,^^11^^,^^12^^,^^15^ Khosrotehrani et al. presented a nomogram for the prediction of prognosis in stage III B/C melanoma patients, using pathology results and age.^24^ Application of this nomogram could further aid in selecting patients for SGD alone. Another preoperative aid in addition to the presented model could be use of the biomarker S-100B. As Kruijff et al. have shown, high serum levels of S-100B are associated with a significantly lower disease-free survival and a trend towards worse melanoma-specific survival (MSS), indicating its potential as a biomarker for clinically occult stage IV disease.^25^^,^^26^ Patients with a low risk of deep pelvic nodal involvement and low S-100B could then undergo SGD alone, with regular control visits to detect early signs of deep pelvic nodal involvement (suspicious nodes on imaging/elevated S-100B). Bearing this in mind, the 16 % FN rate of the presented prediction model may be allowable.

### Limitations

This study was retrospective and was spread over a long timeframe. This entails inevitable alterations and improvement of imaging techniques and clinical practice over time, affecting our results. The prediction model designed for the current study has not been validated internally due to a small sample of patients with positive deep pelvic nodes. It has to be pointed out that this model in its current state is not suited for clinical use as there is still much to be gained from further development and testing. A prospective, multicenter registration study is planned, enabling adequate data collection on all patients undergoing CGD for palpable groin metastases within a relatively small timeframe. Cross-validation of the presented prediction model will be performed and its role in future clinical practice will be further defined. With the proposed prospective study, accuracy of imaging techniques can be determined more adequately.

Regarding the possible additional morbidity of a DGD, although to date no prospective randomized controlled trial (RCT) has been performed to address this, evidence exists that the additional morbidity of DGD in a CGD might be more limited than has been described in the past.^6^^,^^22^ The recently opened Australia and New Zealand Melanoma Trials Group 01.12 Evaluation of Groin Lymphadenectomy Extent For Metastatic Melanoma (EAGLE FM) trial (clinicaltrials.gov identifier NCT02166788) will hopefully provide an answer to this question. This multicenter RCT compares SGD and CGD for melanoma patients with groin metastases and no suspicious PET/CT scan.

As operating time is generally longer in a CGD, there is a potentially higher risk of surgical site infections. In the large, retrospective series of Glarner et al. the number of surgical site infections is indeed significantly higher for CGDs, with an adjusted OR of 2.6.^27^ Once again, to gain more insight into the actual differences in morbidity between SGD and CGD, we will have to await results from the EAGLE FM Trial.

## Conclusions

High LNR, high number of positive inguinal nodes, and inguinal ECE are risk factors for positive deep pelvic nodes in patients with palpable groin metastases of cutaneous melanoma. To date, accurate prediction of deep pelvic nodal status is still suboptimal, hence reliable selection of patients who can be spared a DGD remains difficult. Combined use of preoperative imaging and a preliminary prediction model based on histopathology results of the inguinal (superficial) part of CGD could accurately predict negative deep pelvic nodes in up to 84 % of patients, thereby potentially identifying a group of low-risk patients in whom the extent of surgery might safely be minimized. The risk factors and the prediction model will be further investigated in a prospective, multicenter registry trial for CGDs.
